# Production of tetravalent dengue virus envelope protein domain III based antigens in lettuce chloroplasts and immunologic analysis for future oral vaccine development

**DOI:** 10.1111/pbi.13065

**Published:** 2019-02-19

**Authors:** André van Eerde, Johanna Gottschamel, Ralph Bock, Kristine Eraker Aasland Hansen, Hetron Mweemba Munang'andu, Henry Daniell, Jihong Liu Clarke

**Affiliations:** ^1^ NIBIO – Norwegian Institute of Bioeconomy Research Division of Biotechnology and Plant Health Ås Norway; ^2^ Max Planck Institute of Molecular Plant Physiology Potsdam‐Golm Germany; ^3^ Norwegian University of Life Sciences Faculty of Veterinary Medicine Ås Norway; ^4^ Department of Biochemistry School of Dental Medicine University of Pennsylvania Philadelphia PA USA

**Keywords:** lettuce chloroplast transformation, dengue fever, recombinant EDIII protein, immunogenicity, gastrointestinal digestion analysis, oral vaccine

## Abstract

Dengue fever is a mosquito (*Aedes aegypti*) ‐transmitted viral disease that is endemic in more than 125 countries around the world. There are four serotypes of the dengue virus (DENV 1‐4) and a safe and effective dengue vaccine must provide protection against all four serotypes. To date, the first vaccine, Dengvaxia (CYD‐TDV), is available after many decades’ efforts, but only has moderate efficacy. More effective and affordable vaccines are hence required. Plants offer promising vaccine production platforms and food crops offer additional advantages for the production of edible human and animal vaccines, thus eliminating the need for expensive fermentation, purification, cold storage and sterile delivery. Oral vaccines can elicit humoural and cellular immunity *via* both the mucosal and humoral immune systems. Here, we report the production of tetravalent EDIII antigen (EDIII‐1‐4) in stably transformed lettuce chloroplasts. Transplastomic EDIII‐1‐4‐expressing lettuce lines were obtained and homoplasmy was verified by Southern blot analysis. Expression of EDIII‐1‐4 antigens was demonstrated by immunoblotting, with the EDIII‐1‐4 antigen accumulating to 3.45% of the total protein content. Immunological assays in rabbits showed immunogenicity of EDIII‐1‐4. Our *in vitro* gastrointestinal digestion analysis revealed that EDIII‐1‐4 antigens are well protected when passing through the oral and gastric digestion phases but underwent degradation during the intestinal phase. Our results demonstrate that lettuce chloroplast engineering is a promising approach for future production of an affordable oral dengue vaccine.

## Introduction

Dengue fever is caused by four antigenically distinct dengue virus serotypes (DENV‐1, DENV‐2, DENV‐3 and DENV‐4). Dengue viruses belong to the family Flaviviridae and mainly occur in the tropical and subtropical parts of the world (Calisher *et al*., 1989; Weaver and Vasilakis, 2009). It is estimated that about 3.9 billion people in more than 125 countries are at risk of dengue infection with an annual dengue infection scale of approximately 390 million (Bhatt *et al*., 2013). Of these 390 million infected people, approximately 500 000 need hospital treatment (WHO position report September 2018, Vannice *et al*., 2015, 2016; Wichmann *et al*., 2017). Primary infection with one of the mosquito‐transmitted serotypes usually causes mild dengue fever and provides lifelong immunity to that serotype (Kurane and Ennis, 1992; Simmons *et al*., 2012). Secondary infections with a heterologous serotype result in more life‐threatening and potentially deadly forms of the disease (WHO, 2009) due to antibody dependent enhancement. This phenomenon is associated with cross‐reactive but non‐neutralizing antibodies produced during the first infection (Halstead, 1988), which enhance the uptake of viruses into Fc‐receptor bearing cells during secondary infection (Dejnirattisai *et al*., 2010; Halstead, 2003). This increases the initial virus load in the cells, promotes virus replication and ultimately leads to overwhelming of the immune system, causing symptoms like fluid accumulation, plasma leaking, respiratory distress, severe bleeding and organ impairment (WHO, 2015). Recent outbreaks of dengue fever in the southern provinces of China (Xiong and Chen, 2014; Zhang *et al*., 2014) with over 40 000 reported cases and the occurrence of autochthonous transmissions in the non‐endemic regions of eastern China (Wang *et al*., 2015; Xu *et al*., 2007) show how serious this disease is. A safe, effective and affordable dengue vaccine against the four strains is urgently needed to control the disease and meet the WHO goal of reducing dengue morbidity by at least 25% and mortality by at least 50% by 2020 (www.who.int).

As complete eradication of the mosquito vector is impossible, vaccination seems to be the most promising protection strategy against dengue fever. The co‐circulation of the four dengue virus serotypes in most areas together with the complex pathogenesis have considerably hampered vaccine development (Ghosh and Dar, 2015; WHO, 2015). One dengue vaccine has been licensed, Dengvaxia^®^ (CYD‐TDV, developed by Sanofi Pasteur) and approximately five additional vaccine candidates are in clinical development, with two (developed by NIH/Butantan and Takeda) now in phase III trials (Vannice *et al*., 2016; Wichmann *et al*., 2017). The CYD‐TDV vaccine is a live recombinant tetravalent dengue vaccine that is currently recommended in three doses at 0, 6 and 12 months, but it only has moderate efficacy. The other five vaccine candidates currently under evaluation in clinical trials include other live‐attenuated vaccines, as well as subunit, DNA and purified inactivated vaccine candidates (www.who.int). Development of dengue vaccine that is effective for infants and children is needed to reduce the dengue burden.

A promising alternative approach is the development of a recombinant protein‐based vaccine able to stimulate the protective immune system in a balanced way. The domain III of the dengue virus envelop protein (EDIII) protrudes from the virus surface to facilitate binding to the host cell surface receptor (Crill and Roehrig, 2001) and mediates membrane fusion (Allison *et al*., 2001). This approximately 100 amino acid long domain has become the focus of subunit vaccine development (Guzman *et al*., 2010), because it contains a number of epitopes that elicit serotype specific neutralizing antibodies (Chin *et al*., 2007; Megret *et al*., 1992). Different expression systems have been used so far to express recombinant dengue antigens based on the whole envelope protein or the EDIII domain (Batra *et al*., 2010a; Cardoso *et al*., 2013; Clements *et al*., 2010; Ivy *et al*., 2000; Martínez *et al*., 2010; McDonald *et al*., 2009; Saejung *et al*., 2007; Simmons *et al*., 1999; Srivastava *et al*., 2000). Combination of the EDIIIs of the four dengue virus serotypes resulted in a tetravalent fusion protein capable of stimulating the production of virus‐neutralizing antibodies against all four serotypes in mice (Batra *et al*., 2010b; Etemad *et al*., 2008). Since a simultaneous and balanced immune response against all four serotypes is essential (Hombach *et al*., 2005), a vaccine candidate based on the recombinant tetravalent fusion protein appears the best solution.

Genetic engineering of the plastid genome of crops has evolved over the past decades into a promising approach for the production of high‐value products such as biopharmaceuticals, industrial enzymes and diagnostic reagents (Bock, 2015; Clarke and Daniell, 2011; Maliga and Bock, 2011). The distinct characteristics of chloroplast transformation such as site‐specific transgene integration (Daniell, 2006), the absence of epigenetic gene silencing and position effects (Daniell *et al*., 2001; Rigano *et al*., 2009; Verma *et al*., 2010), stacking of transgenes into operons (Bock, 2013) and the excellent biosafety profile of transplastomic plants offer great potential in plant biotechnology. Significant interest in producing recombinant proteins in plastids of crop species with edible parts has resulted in transplastomic carrots (Kumar *et al*., 2004), tomato (Ruf *et al*., 2001; Zhou *et al*., 2008), potato (Sidorov *et al*., 1999), soybean (Dufourmantel *et al*., 2004; Moravec *et al*., 2007), cauliflower (Nugent *et al*., 2006), eggplant (Singh *et al*., 2010), cabbage (Liu *et al*., 2007) and sugar beet (De Marchis *et al*., 2009). Edible crops offer the potential of oral delivery of therapeutical proteins, resulting in much reduced downstream protein processing costs (Kwon *et al*., 2013; Streatfield, 2006).

Since the first report of transplastomic lettuce plants, the evaluation of different integration sites and transformation strategies have led to the successful expression of several recombinant proteins in lettuce plastids (Boyhan and Daniell, 2011; Davoodi‐Semiromi *et al*., 2010; Ichikawa *et al*., 2010; Kanagaraj *et al*., 2011; Kanamoto *et al*., 2006; Maldaner *et al*., 2013; Ruhlman *et al*., 2007). Most importantly, lettuce is the only system so far that has been shown to be feasible for commercial scale production of clinical grade biopharmaceuticals (Su *et al*., 2015). In large animal and toxicology studies (Herzog *et al*., 2017) biopharmaceuticals were shown to be stable for up to 30 months in lyophilized lettuce cells when stored at ambient temperature, without loss of activity. Furthermore, protection of biopharmaceuticals upon passage of the stomach by bioencapsulation within plant cells has been repeatedly demonstrated. When reaching the gut, commensal bacteria degrade plant cell walls, thereby releasing the protein drugs and facilitating delivery to the immune and/or circulatory systems (Kwon and Daniell, 2016; Xiao *et al*., 2016). Orally administered plant‐produced antigens can stimulate mucosal IgA and serum IgG production (Lee *et al*., 2015) and antigen fusion to a transmucosal carrier like the cholera toxin B subunit (CTB) improves the efficiency of antigen delivery to the immune system (Chan and Daniell, 2015; Davoodi‐Semiromi *et al*., 2010). A further recent example is provided by the production of a plant‐based oral booster vaccine against polio virus, for use in a routine vaccination strategy with a booster dose applied after at least 6 months. The efficacy of the vaccine in eliciting mucosal and humoural immune responses could be demonstrated (Xiao and Daniell, 2017; Xiao *et al*., 2016; Zhang *et al*., 2017).

In the current study, we have expressed a tetravalent fusion protein EDIII‐1‐4 in lettuce chloroplasts, performed an *in vitro* gastrointestinal digestion study and tested the stability of the bioencapsulated recombinant protein throughout the human upper digestive system. Our results showed (i) the successful production of tetravalent EDIII antigen (EDIII1‐4) in lettuce chloroplasts; (ii) molecular analyses of transplastomic EDIII‐1‐4 ‐expressing lettuce lines; (iii) immunoblotting analysis of EDIII‐1‐4 accumulation in lettuce; (iv) immunological assays in rabbits with tetravalent EDIII‐1‐4 antigens; and (v) the results from the *in vitro* gastrointestinal digestion analysis including oral phase, gastric phase and intestinal phase. Our results indicate that lettuce chloroplast engineering represents a promising approach for the production of a safe and affordable oral dengue vaccine and have generated new information for the dengue vaccine research community.

## Results

### Generation and characterization of dengue virus EDIII‐1‐4 producing transplastomic lettuce

**Figure 1:**
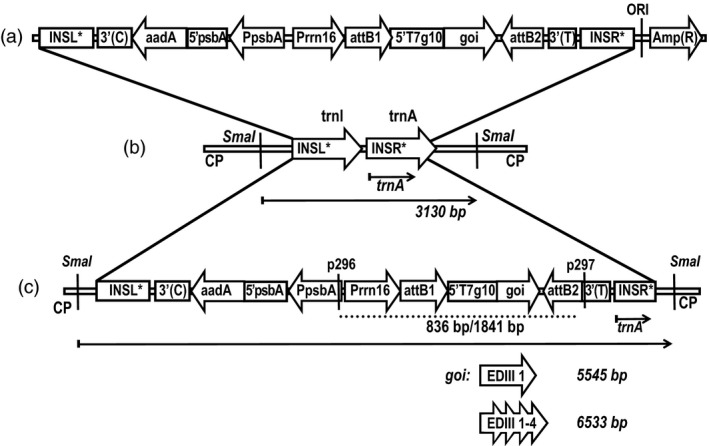
Schematic representation of the expression vectors for the generation of transplastomic lettuce plants: (a) The final lettuce‐specific plastid transformation vector pEXP‐PN‐goi‐L. (b) wild‐type lettuce plastid genome (CP). (c) lettuce plastid genome with integrated transgene expression cassettes for *ediii‐1* and *ediii‐1‐4*, separately. The Southern blot probe (*trnA*) is shown as an arrow and the expected SmaI fragments are shown as arrows with their sizes indicated next to the respective goi. aadA: spectinomycin resistance gene; Amp(R): ampicillin resistance gene; attB1/attB2: Gateway^®^ recombination sites; INSL*/INSR*: lettuce‐specific left/right flanking regions; trnI/trnA: sequences coding for tRNA‐Ile/tRNA‐Ala; EDIII 1/1‐4: transgene coding sequence including a hexa‐his‐tag; PsbA: tobacco *psbA* promoter (Staub and Maliga, 1993); Prrn16: tobacco rrn16 PEP+NEP promoter (Ye et al., 2001); 3′(C): 3′UTR of *Chlamydomonas rbcL* gene; 5′psbA: 5′UTR of tobacco *psbA* gene; 3′(T): 3′UTR of tobacco *rbcL* gene; ORI: bacterial origin of replication. p296/p297: primer used for PCR (the corresponding PCR products are shown as dotted lines and the sizes are given for both transgenes).

In order to produce a dengue antigen that covers all four dengue virus serotypes, transplastomic plants expressing the tetravalent antigen EDIII‐1‐4 (Gottschamel *et al*., 2016) in the chloroplast were generated. To compare expression levels and help ascertain the stability of the linker regions in the tetravalent antigen, a transplastomic line expressing only the EDIII from DENV‐1 was also generated. First, a lettuce plastid transformation vector pDEST‐PN‐L was constructed by insertion of the *aadA* expression cassette and the Gateway^®^ RfA between lettuce‐specific flanking regions for integration into the plastid genome by homologous recombination. The vectors pEXP‐PN‐ediii‐1‐L and pEXP‐PN‐ediii‐1‐4‐L (Figure 1a) for lettuce plastid transformation were then obtained by Gateway^®^ cloning of the sequences for ediii‐1 and ediii‐1‐4 into the lettuce‐specific pDEST‐PN‐L. Integration by homologous recombination into the intergenic spacer region between the *trnI* and *trnA* genes results in transplastomic plants carrying the transgene expression cassettes within the IR region of the lettuce plastid genome (Figure 1b,c).

**Figure 2:**
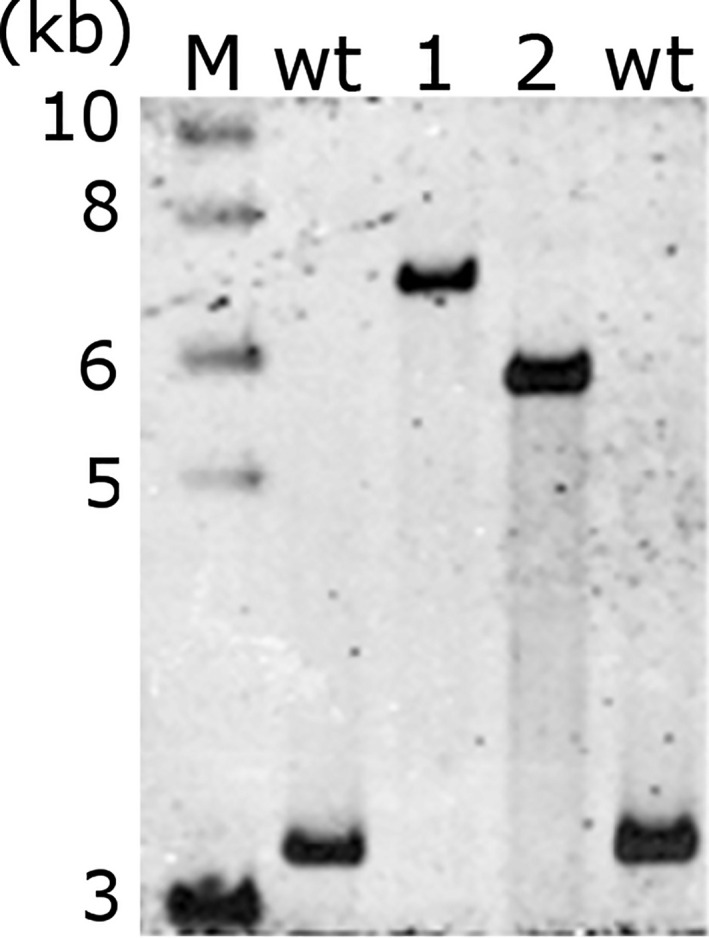
Southern blot analysis of tetravalent EDIII‐1‐4 and monovalent EDIII‐1 lines. DNA isolated from regenerated plant lines and the wild‐type (wt) was probed using a 665 bp DIG‐labelled probe that binds inside the *trnA* region (INSR) of the plastid genome. The expected fragment sizes after SmaI digestion are 6533 bp (for S12‐PN‐EDIII‐1‐4), 5545 bp (for S16‐PN‐EDIII‐1) and 3130 bp (for wild‐type plants). The positions of restriction sites, probe position and the sizes of expected Southern blot bands are indicated in Figure 1. M: 1 kb DNA ladder, (NEB).

The two transformation constructs were introduced into plastids by particle bombardment. Antibiotic‐resistant shoots developing from callus tissue on RMOP plant regeneration medium containing spectinomycin were tested for transgene integration by PCR. Presence of the transgenic sequences in the plastid genome was shown by PCR products corresponding to ediii‐1‐4 (1841 bp) and ediii‐1 (836 bp) (Figure 2a). The transplastomic plant lines (S12‐PN‐EDIII‐1‐4 and S16‐PN‐EDIII‐1 respectively) were further characterized by Southern blot analysis. The homoplastomic state of both plant lines was verified by the presence of only the 5545 bp fragment (in S16‐PN‐EDIII‐1) or the 6533 bp fragment (in S12‐PN‐EDIII‐1‐4) in transformed plants, compared to the 3130 bp fragment diagnostic of the wild‐type plastid genome (Figure 2b) after digestion of total plant DNA with *Sma*I.

**Figure 3:**
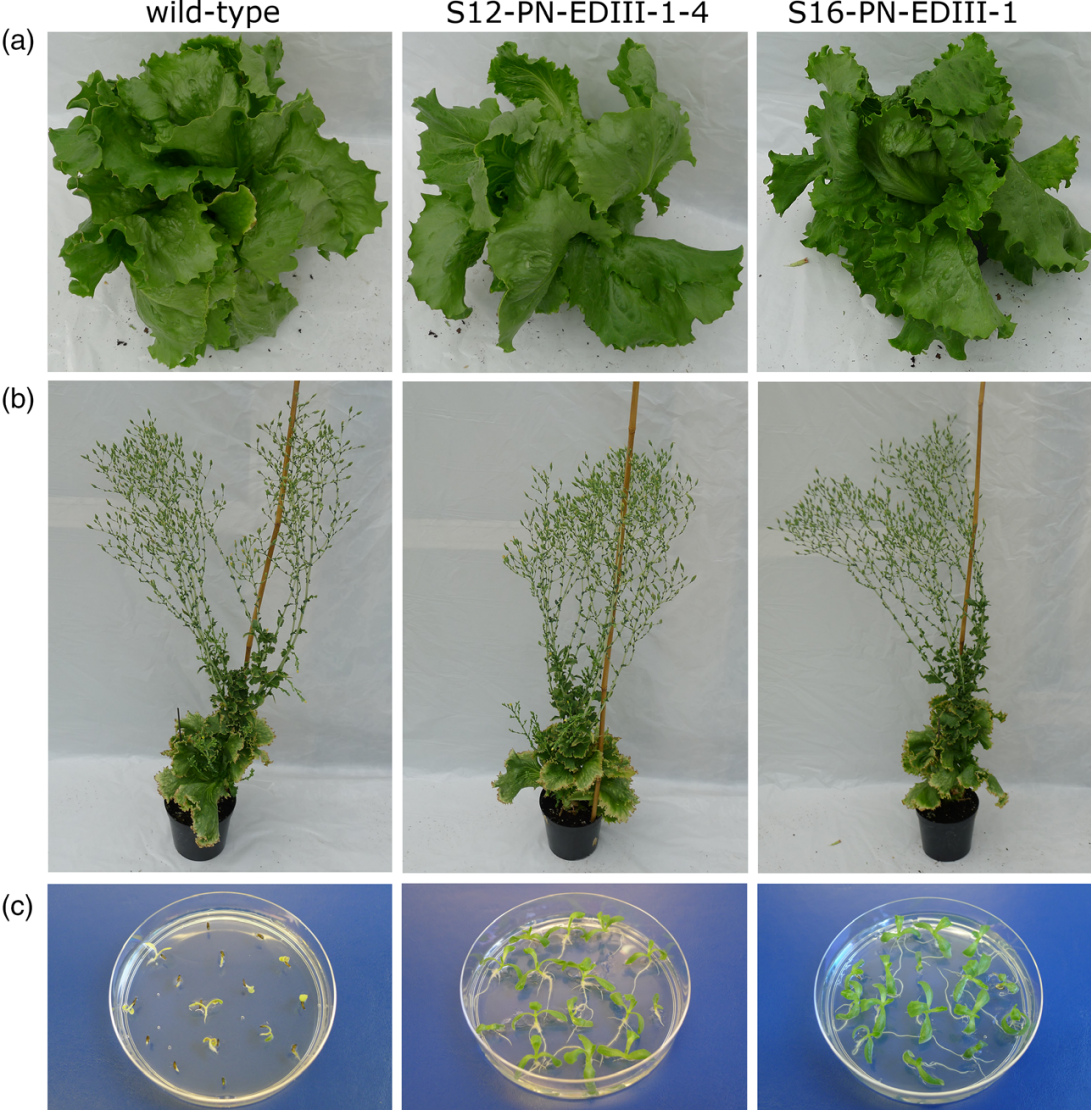
Phenotype of transplastomic lettuce plants and inheritance assays. (a) Plants growing in the greenhouse. (b) Flowering plants. (c) One‐week‐old seedlings obtained from transplastomic plants and wild‐type seeds germinated on spectinomycin (30 mg/L) containing medium.

No phenotypic alterations were visible on transplastomic plants growing to maturity in the greenhouse (Figure 3a) and flower set and seed development was normal. Plants were grown to full maturity (Figure 3b) and seeds harvested from transgenic plants were germinated on spectinomycin‐containing medium. The homogenous green phenotype of the seedlings proved the absence of segregation of the antibiotic resistance gene in the F1 generation (Figure 3c) provided additional proof of transgene integration into the plastid genome and complete elimination of wild‐type copies of the (polyploid) plastid genome.

### Expression of EDIII‐1‐4 and EDIII‐1 antigens

**Figure 4:**
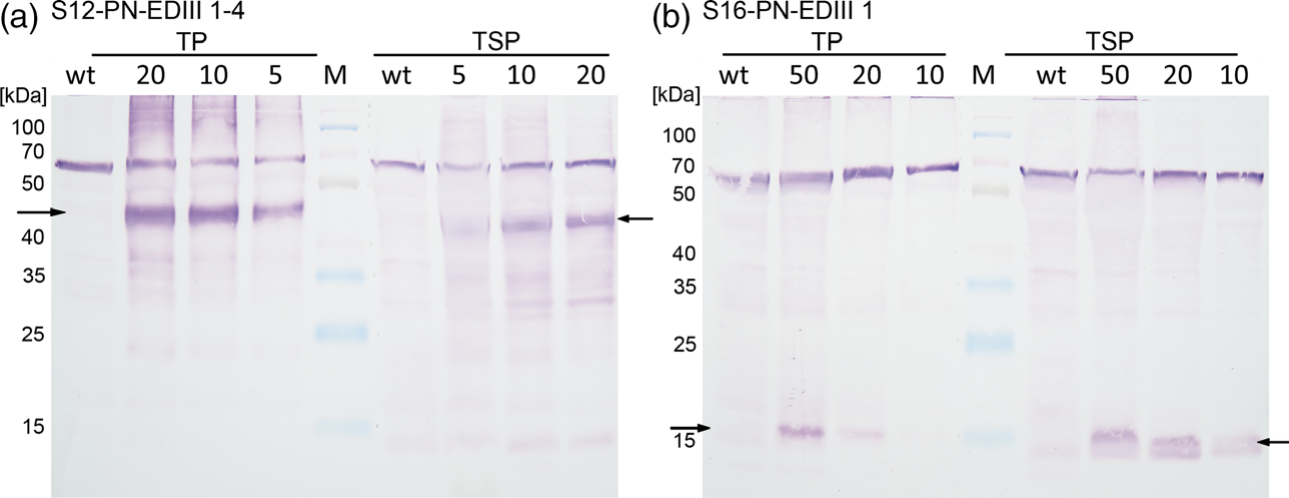
Western blot analysis of EDIII antigen accumulation in transplastomic lettuce plants. (a) TP and TSP isolated from plant line S12‐PN‐EDIII‐1‐4. (b) TP and TSP isolated from plant line S16‐PN‐EDIII‐1. The amount of TSP/TP loaded is given above the respective lane in μg. Twenty microgram TP/TSP was loaded for the wild‐type in a), while 50 μg TP/TSP was loaded for the wild‐type in b). The arrows indicate the 47 kDa EDIII‐1‐4 and the 13 kDa EDIII‐1. M: spectra multicolour broad range protein ladder (Thermo Scientific, molecular weight of the marker bands indicated in kDa). The band migrating at 70 kDa results from non‐specific binding of the antibody to a plant protein of unknown identity (Gottschamel *et al*., 2016).

In order to assess whether the antigens were produced and accumulated stably in lettuce chloroplasts, total protein (TP) and total soluble protein (TSP) were isolated from plant lines growing in the greenhouse and quantified by BCA and Bradford assays respectively. Immunoblot analysis performed with an anti‐dengue antibody detected both the 47 kDa EDIII‐1‐4 and the 13 kDa EDIII‐1 in the respective transplastomic lettuce lines, in both the TP and TSP samples (Figure 4). The single‐domain antigen EDIII‐1 accumulated to lower levels (Figure 4b) and this line was not studied further. The tetravalent antigen EDIII‐1‐4 was only detected as a single band corresponding to the full‐length antigen, indicating that the linker regions are stable in the chloroplast and no significant proteolytic cleavage occurs. The EDIII‐1‐4 accumulation was quantified in TP extracts by using purified *E. coli*‐produced tetravalent antigen (Gottschamel *et al*., 2016) as a standard. These assays (Figure S1) established that the EDIII‐1‐4 fusion protein was present at 3.45% ± 0.05 of the TP content in the transplastomic lettuce plants.

### Immunogenicity of lettuce‐produced EDIII‐1‐4 in rabbits

**Figure 5:**
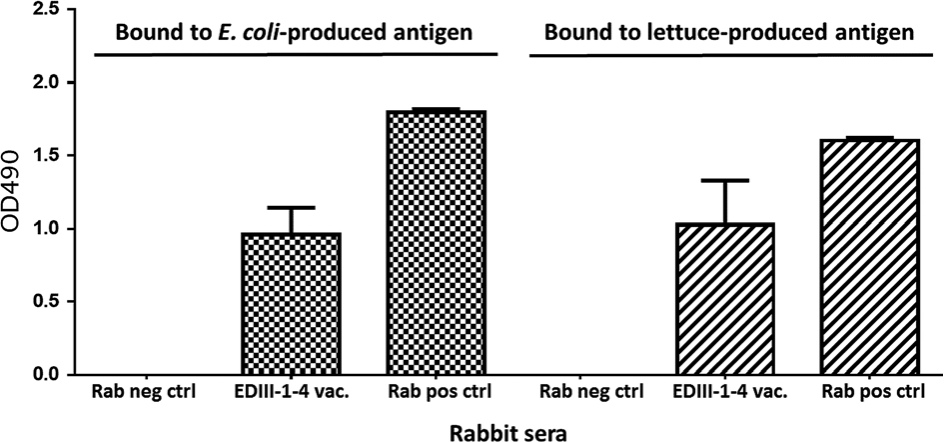
Immunogenicity of lettuce‐produced tetravalent EDIII 1‐4. Sera of a rabbit immunized with lettuce‐produced EDIII‐1‐4 antigen at 63 days post vaccination (EDIII‐1‐4) were tested for binding to *E. coli*‐produced EDIII‐1‐4 antigen and lettuce‐produced EDIII‐1‐4 antigen. Rab neg ctrl is serum from non‐vaccinated rabbit; Rab Pos Ctrl represents a rabbit polyclonal antibody against a synthetic EDIII peptide (Gottschamel *et al*., 2016) used as a positive control. All sera were used in a 1 : 50 dilution.

Next, we wanted to test the immunogenicity of the lettuce‐produced EDIII‐1‐4 antigens. To this end, the fusion protein was isolated from the transplastomic lettuce line S12‐PN‐EDIII‐1‐4 under native conditions and used for immunization of one rabbit. Rabbit serum was analysed by an enzyme‐linked immunosorbent assay (ELISA), where binding to lettuce‐produced and *E. coli*‐produced EDIII‐1‐4 was assayed. Injection of lettuce‐produced dengue antigen EDIII‐1‐4 elicited antibodies detectable by ELISA at 9 weeks post vaccination (Figure 5). It is interesting to note that the rabbit serum recognized the lettuce‐produced antigen (mean antibody titre 1.0235 OD_490_, SD = 0.4305) and the *E. coli*‐produced EDIII‐1‐4 antigen (mean antibody titre 0.9598 OD_490_, SD = 0.2565) equally well, confirming that the antigens are specifically recognized by the serum. The results of our immunological analyses show that rabbit vaccination with lettuce‐produced EDIII‐1‐4 antigen elicits a specific immune response.

### Stability of lettuce‐encapsulated EDIII‐1‐4 in a gastrointestinal tract model

**Figure 6:**
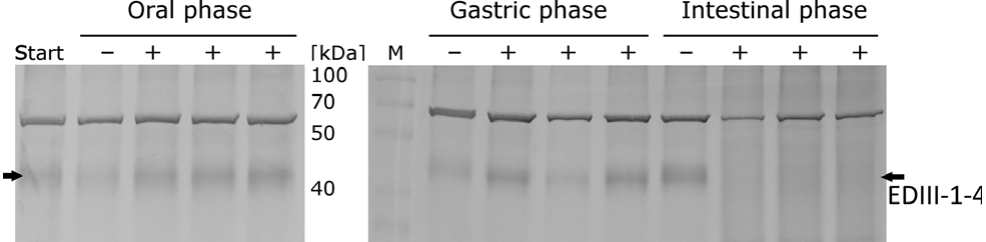
*In vitro* gastrointestinal digestion analysis of the lettuce chloroplast‐derived EDIII‐1‐4 tetravalent antigen. Western blot analysis of proteins isolated from samples taken for each phase of the *gastrointestinal digestion* analysis are shown. Start: untreated lettuce material; − samples treated with only buffer; + samples treated with the digestive enzymes. Each phase was analysed in three replicates with enzymes and one blank sample without enzymes as control. Thirty microgram TP was loaded for each sample. The band migrating at 70 kDa results from non‐specific binding of the antibody to a plant protein of unknown identity.

Simulated gastrointestinal digestion is widely employed in food science, nutritional studies and pharmaceutical research, as conducting human trials is costly, resource intensive and ethically disputable. The methodology is based on mimicking physiological conditions *in vitro*, taking into account the presence and physiological concentrations of digestive enzymes as well as the pH, ionic milieu and digestion time, among other factors (Minekus *et al*., 2014). Simulated digestion of lettuce chloroplast‐derived EDIII‐1‐4 tetravalent antigen through the oral, gastric and small intestinal phases (Figure 6) showed that EDIII‐1‐4 tetravalent antigen was well protected when passing through the oral phase and the gastric phase, but showed degradation after passing through the intestinal phase. The latter is consistent with the (desired) release of the antigen from the chloroplasts in the intestine, where it is intended to stimulate mucosal immunity.

## Discussion

Despite over 70 years of efforts to combat the disease, dengue fever is still a major health threat in significant parts of the world. In view of the expected further spread of mosquito‐borne diseases with climate change, the need for dengue vaccine development becomes even more pressing (Pang and Loh, 2017). Efficacy and safety remain the main challenges (Wichmann *et al*., 2017; www.who.int). Clinical studies with the licensed Dengvaxia^®^ (CYD‐TDV, a live‐attenuated, recombinant tetravalent vaccine employing the attenuated YF virus 17D vaccine strain as the replication backbone) have been further investigated since 2015. The new analyses from the long‐term safety follow‐up indicated that overall the population level benefit of vaccination remains favourable, but the vaccine performs differently in seropositive versus seronegative individuals. Vaccine efficacy (VE) confirmed that symptomatic dengue was high among inferred baseline seropositive participants ≥9 years of age: 76% (95% CI: 63.9, to 84.0), but was much lower among baseline seronegative participants: 38.8% (95% CI: −0.9 to 62.9) in the first 25 months after the first dose of vaccine (www.who.int, Wichmann *et al*., 2017). These data demonstrated long‐term protection in seropositive individuals. However, at the same time, the studies also revealed an excess of hospitalized and severe dengue cases in seronegative vaccine recipients compared to seronegative non‐vaccinated individuals and there was an increase in the number of young vaccinated children hospitalized within 3 years after the start of vaccination (Wichmann *et al*., 2017). These data provide a strong incentive to look for improved vaccination strategies. A safe, effective and affordable vaccination strategy shall include careful screening pre‐vaccination, the affordability of both CYD‐TDV and screening tests and assessing the need and feasibility of oral booster dengue vaccine (Wichmann *et al*., 2017). Vaccination as part of an integrated dengue prevention and control strategy remains a high priority (Wichmann *et al*., 2017). More efforts must be invested into the development of safe, effective and affordable dengue vaccines that, ideally, should be independent of the serostatus (Wichmann *et al*., 2017) and employ an oral vaccine and/or an oral booster vaccine produced in an edible plant such as lettuce.

We have previously reported synthesis of an EDIII‐1‐4 tetravalent antigen‐based subunit dengue vaccine in tobacco chloroplasts (Gottschamel *et al*., 2016). In the present study, we have successfully expressed the tetravalent antigen in chloroplasts of the edible crop lettuce, assayed its stability upon passage through the gastrointestinal tract and determined its antigenicity in experimental animals. Further efforts in producing other dengue antigens in plants have also been reported (Kanagaraj *et al*., 2011; Maldaner *et al*., 2013). Our work adds to a growing number of studies in plant molecular farming that have demonstrated that production of vaccines and antibodies in plants is a very attractive alternative to traditional production systems. Plants offer a number of unique advantages over more traditional systems (Bock, 2015; Clarke and Daniell, 2011; Peyret and Lomonossoff, 2015), including cost‐effective production of recombinant proteins at large scale (due to the very low production costs of plant biomass and the easily scalable production levels), the low risk of contamination with human pathogens and the possibility of oral delivery. The latter eliminates the need for expensive downstream processing (usually about 80% of the production cost of pharmaceuticals and vaccines), makes vaccine administration simple and safe (Chan and Daniell, 2015; Clarke *et al*., 2017) and provides stability of antigens at room temperature, a highly desired property of vaccines.

Protein drugs expressed within plant cells can be protected from acids and enzymes in the stomach (Chan and Daniell, 2015). To assess the stability of the tetravalent EDIII‐1‐4 antigens produced in chloroplasts after oral delivery, we have conducted *in vitro* gastrointestinal tract assays. The simulated digestion of lettuce chloroplast‐derived EDIII‐1‐4 tetravalent antigen through the oral, gastric and small intestinal phases has shown that the antigen was well‐protected when passing through the oral and gastric phases and showed degradation upon passing through the intestinal phase (Figure 6). The latter is not only consistent with the expected release of the protein from the plant cells in the intestine, but also desirable to expose the antigen to the mucosal immune system.

The prime‐and‐boost based vaccination strategy has been exploited with plant‐made boost vaccines against HIV and polio virus (Chan *et al*., 2016; Lindh *et al*., 2014). The same strategy could be applied to dengue fever, given that, with the current vaccine, the immune response varies greatly among different people, age groups and regions. Moreover, the incidence of children developing dengue infection within 3 years after vaccination in one of the CYD‐TDV dengue vaccine clinical trials (Wichmann *et al*., 2017) suggests the potential benefit of a booster vaccine for young children. Together with a previous report (Chan and Daniell, 2015), our study suggests that the edible crop lettuce is ideal for the production of oral boost vaccines. This is not only because lettuce can be eaten raw, but also because the edible parts of the plants are the leaves. A number of studies have shown that expression of plastid genes and transgenes in non‐green tissues (including fruits and tubers) is much lower than in photosynthetically active leaf tissue (Kahlau and Bock, 2008; Valkov *et al*., 2009). A plant‐made dengue boost vaccine could contribute to affordable dengue treatment and reduce the burden on the health sector in developing countries. Lettuce chloroplast genome engineering could contribute to an oral boost dengue vaccine in the future.

In conclusion, we have demonstrated in the present study the successful production of the tetravalent EDIII dengue virus antigen (EDIII‐1‐4) in lettuce chloroplasts. Homoplastic transplastomic lettuce lines expressing EDIII‐1‐4 tetravalent dengue vaccine antigen were obtained and homoplasmy was verified by Southern blot analysis. The expressed tetravalent EDIII‐1‐4 antigen was detected in immunoblotting and quantified. Immunological assays showed immunogenicity in rabbits. Our *in vitro* gastrointestinal digestion analysis with EDIII‐1‐4‐producing lettuce has shown that EDIII‐1‐4 antigens were well protected when passing through the oral and gastric phases. Our results suggest that lettuce chloroplast engineering is a promising approach for the future production of a safe, affordable and effective oral dengue boost vaccine that can contribute to the control and management of dengue infection globally.

## Methods

### Vector construction

The lettuce‐specific plastid transformation vector was obtained by Gateway^®^ cloning (Gottschamel and Lössl, 2016;). For the intermediary vector pMA‐lettuce, the sequences corresponding to the *trnI* and *trnA* region of the *Lactuca sativum* plastid genome (Ruhlman *et al*., 2007) flanked by *Kpn*I and *Sac*II restriction sites were custom synthesized and introduced into the company's standard backbone vector (GeneArt, Germany). In order to create pDEST‐PN‐L, the complete Gateway^®^ cloning cassette together with the *aadA* expression cassette was excised from pDEST‐PN‐T (Gottschamel *et al*., 2013) and inserted into pMA‐lettuce using the restriction enzymes *Kpn*I and *Sac*II. The sequence of the synthetic fusion gene (*ediii*‐1‐4) consists of all four DENV‐EDIII sequences (order: *ediii*‐1, *ediii*‐3, *ediii*‐4, *ediii*‐2) linked by penta‐glycine linkers (Etemad *et al*., 2008; Gottschamel *et al*., 2016). The *ediii‐1‐4* and *ediii‐1* sequences were codon optimized for lettuce plastids and synthesized by GeneArt (Germany). The synthesized sequences have a T7g10 leader sequence and a 15 nucleotide downstream box (Herz *et al*., 2005), a C‐terminal 6xHis‐tag and are flanked with *attB1*/*attB2* Gateway^®^ recombination sites. The transgene encoding sequences were first introduced into pDONR™221 by a BP reaction yielding the intermediary vectors pEntry‐*ediii*‐1‐4 and pEntry‐*ediii*‐1 and then transferred by an LR reaction into pDEST‐PN‐L resulting in the final lettuce‐specific plastid transformation vectors pEXP‐PN‐ediii‐1‐4‐L and pEXP‐PN‐ediii‐1‐L respectively. The PCR Cloning Kit with Gateway^®^ Technology, pDONR^™^ 221 and the Gateway^®^ LR Clonase^®^ Enzyme mix were purchased from Life Technologies (Carlsbad, CA) and the Gateway^®^ BP and LR reactions (Karimi *et al*., 2002) were carried out as described in the manufacturer's protocol.

### Plant growth, transformation and regeneration

*Lactuca sativa* cv. Barkley plants were grown *in vitro* from surface sterilized seeds on solid MS medium (Murashige and Skoog, 1962) containing 20 g/L sucrose. Leaves from aseptically grown lettuce plants were bombarded with 0.6 μm gold‐microcarriers coated with plasmid DNA using a Bio‐Rad Biolistic PDS‐1000/He gun (Daniell, 1997; Svab and Maliga, 1993) Several independently transformed plant lines were subjected to three additional regeneration rounds on RMOP medium (Svab and Maliga, 1993; Verma *et al*., 2008) containing spectinomycin. Regenerated shoots were rooted on MS medium containing spectinomycin to maintain the selection pressure. Rooted, homoplastomic plants were transferred to soil and grown to maturity in the greenhouse under standard conditions. Inheritance assays on spectinomycin‐containing MS medium were performed with the harvested seeds.

### Southern blot analysis

Plant DNA was isolated by the CTAB procedure (Murray and Thompson, 1980) from wild‐type plants and transplastomic plant lines after three rounds of regeneration on spectinomycin‐containing medium. Ten microgram of plant DNA was digested with *Sma*I, separated by electrophoresis in a 1% agarose gel and transferred onto a positively charged nylon membrane (Carl Roth GmbH, Karlsruhe, Germany) by capillary action using the semi‐dry transfer method. The probe binding inside the *trnA* region was amplified from lettuce wild‐type DNA by PCR (primers 5′‐GGAGGTAGGATGGGCAGTTG‐3′ and 5′‐GGACTCGAACCGCTGACATC‐3′). The probe was purified by agarose gel electrophoresis, followed by extraction of the fragment of interest from excised gel slices with the NucleoSpin gel and PCR Clean‐up Kit (Machery‐Nagel, Düren, Germany). The probe was DIG labelled using the DIG‐High Prime DNA Labeling and Detection Starter Kit II (Roche, Basel, Switzerland), according to the manufacturer's instructions. After immobilization of the DNA to the membrane, hybridization with the corresponding DIG labelled‐probe and incubation of the membrane with the HRP conjugated anti‐DIG antibody, the chemiluminescence signal was detected by exposure to X‐ray film. One homoplastomic plant line per construct (S12‐PN‐EDIII‐1‐4 and S16‐PN‐EDIII‐1) was chosen for further analysis.

### Protein extraction, Western blot analysis and protein quantification

Protein extraction and Western blot analysis was done as described previously (Gottschamel *et al*., 2016). Briefly, total soluble protein (TSP) was extracted by mixing frozen and grinded leaf samples in TSP extraction buffer [100 mm NaCl, 10 mm EDTA, 200 mm Tris‐HCl pH 8, 0.05% Tween‐20, 0.1% SDS, 14 mm β‐mercaptoethanol, 200 mm sucrose and 1× cOmplete protease inhibitor (Roche)] for 5 min on ice, followed by centrifugation at 15 000 ***g*** for 10 min at 4 °C and collecting the supernatant. The TSP concentration was determined with the Bradford assay (Bio‐Rad, Hercules, CA) using known concentrations of BSA as the standard. Total protein (TP) was isolated from leaf samples by the phenol extraction method (Cahoon *et al*., 1992). Frozen ground leaf samples were homogenized in TP extraction buffer [0.7 m sucrose, 0.5 m Tris‐HCl pH 9.4, 50 mm EDTA, 0.1 m KCl, 2% β‐mercaptoethanol, 1× cOmplete protease inhibitor (Roche, Switzerland)]. After addition of 1 vol. phenol, short vortexing and centrifugation at 13 000 r.p.m. for 10 min at 4 °C, the upper green phase was recovered and proteins were precipitated by addition of 0.1 m NH_4_OAc in methanol and overnight incubation at −20 °C. After centrifugation, the protein pellet was washed, air‐dried and dissolved in 1% SDS. The protein concentration was determined with the BCA Protein Assay Kit (Thermo Scientific, Waltham, MA) using known concentrations of BSA as the standard. Denatured protein samples were separated by electrophoresis in 12% SDS‐polyacrylamide gels and transferred to nitrocellulose membranes (Hybond‐ECL; GE Healthcare, Chicago, IL). The membranes were incubated with TBS‐T (20 mm Tris‐HCl pH 7.6, 137 mm NaCl, 0.1% Tween‐20,) containing 0.5% BSA as blocking buffer and subsequently treated with the primary and the secondary antibody diluted in TBS‐T. The recombinant proteins were detected with the 1:1000 diluted polyclonal anti‐dengue antibody produced in rabbits against amino acid sequence KFKVVKEIAETQHGT (by Davids Biotechnology, Regensburg, Germany), the 1:10 000 diluted anti‐rabbit‐IgG‐AP secondary antibody (Promega, Fitchburg, WI) and colorimetric reaction using the AP color development Kit (Bio‐Rad). Recombinant EDIII‐1‐4, expressed in *E.coli* (Gottschamel *et al*., 2016) and purified with the HisPur Cobalt resin (Life Technologies) under denaturing conditions served as a positive control.

Protein quantification was done by Western blot analysis. Samples with known quantities of the purified *E. coli*‐produced antigen were loaded together with TP extract samples. Blotting was done as described above, but as secondary antibody 1 : 10 000 diluted anti‐rabbit‐IgG‐HRP secondary antibody (Promega) was used. Blot development was done with ECL Prime Western blotting detection reagent (GE Healthcare) and blots were imaged using an Azure C400 imaging system (Azure Biosystems, Dublin, CA) and analysed with ImageJ (NIH, Bethesda, MD). Experiments were repeated four times.

### Immunological studies

The study was performed at the Section for Experimental Biomedicine at The Norwegian University of Life Sciences, Norway. The unit is licensed by the Norwegian Animal Research Authority (NARA) (http://www.mattilsynet.no/dyr_og_dyrehold/dyrevelferd/forsoksdyr/) and accredited by the Association for Assessment and Accreditation of Laboratory Animal Care (www.aaalac.org). The study was approved by the unit's animal ethics committee (Institutional Animal Care and Use Committee/IACUC) and NARA.

### Animal model

The rabbit used in this study was a female New Zeeland White SPF (Harlan Laboratories, Horst, Netherlands). It had 3 weeks of acclimation at the animal facility before starting the immunization. The rabbit was housed and taken care of according to the requirements in the European Union Directive 2010/63/EU and Norway's own regulation based on the EU directive ‘The regulation on use of animals in research FOR‐2015‐06‐18‐761’.

### Tetravalent dengue antigen EDIII‐1‐4 purification and immunization

The EDIII‐1‐4 tetravalent antigen was purified from lettuce line S12‐PN‐EDIII‐1‐4. Frozen leaves were ground to powder, mixed with an equal volume of extraction buffer [0.05 m Tris pH 9.2, 0.5 m NaCl, 0.1% (v/v) Tween 20, 15 mm β‐mercaptoethanol] and incubated on ice for 20 min. The mixture was filtered through four layers of Miracloth (Merck, Darmstadt, Germany) and the filtrate was centrifuged at 25 000 *g* for 40 min at 4 °C. Imidazole pH 9 to a final concentration of 20 mm was added to the supernatant, which was then mixed with NiNTA agarose beads (Qiagen, Hilden, Germany) and incubated under mixing for 3 h at 22 °C. The beads were collected by low‐speed centrifugation and washed with buffer (20 mm imidazole pH 8.6, 0.5 m NaCl, 0.1% Tween 20) and subsequently eluted with elution buffer (0.3 m imidazole pH 8.6, 0.5 m NaCl, 0.1% Tween 20). The buffer was exchanged to 20 mm Tris pH 9, 0.15 m NaCl, 0.1% Tween 20 and the protein was concentrated to 0.2 mg/mL by ultracentrifugation using a 10 kDa MWCO Microsep device (Pall, Westborough, MA).

Injections were done by a veterinarian with FELASA C certification. The rabbit was given one immunization injection and three booster injections. The first immunization was a mixture of 0.5 mL solution containing 0.1 mg antigen and 0.5 mL Freund`s Adjuvant Complete, whereas all three booster injections contained the same amount of antigen but were instead prepared with 0.5 mL Freund`s Adjuvant Incomplete (both from Sigma‐Aldrich, Oslo, Norway). The first booster was given 2 months after the first immunization. All subsequent booster injections were given in 2‐week intervals. The solution was mixed right before injection in two coupled 2 mL Omnifix Luer Lock syringes and then injected with a 23 G Terumo needle (all from Jan F. Andersen, Jevnaker, Norway). All injections were given divided on 5‐10 places. Blood samples were collected before the first and after the last injection, to analyse the antibodies. The blood was taken from the ear artery with a 4 mL Vacuette Serum container and a 0.7 × 25 mm needle (Jan F. Andersen, Jevnaker, Norway). Both the injections and the blood samples were done while the rabbits were sedated with Hypnorm (Fentanyl 0.315 mg/mL and Fluanison 10 mg/mL) 0.03 mL/kg iv. Two weeks after the last booster injection both rabbits were terminally bled under full anaesthesia. They were premedicated with Domitor (Medetomidin 1 mg/mL) 0.1 mL/kg im and anaesthetized with a Zoletil Mix [Zoletil dry matter, 10 mL Rompun (Xylazin 20 mg/mL) and 0.75 mL Torbugesic (Butorphanol 10 mg/mL)] (all from VESO Pharmacy, Oslo, Norway) 0.1 mL/kg im. The blood was taken via heart puncture with a 10 mL Vacuette Serum container and a 0.7 × 25 mm needle (Jan F. Andersen, Jevnaker, Norway). After the bleeding, the rabbits were sacrificed with Penthobarbithal injection to the heart and opening of the thorax.

### Enzyme‐linked immunosorbent assay (ELISA)

ELISA was carried out by coating two plates with a commercial anti‐dengue mouse monoclonal antibody (3H5 against dengue type 2, Temecula, CA) at a dilution of 1 : 1000. The coated plates (NUNC, Denmark) were incubated at 4 °C overnight. After incubation, the plates were washed three times with phosphate buffered saline containing 1% Tween 20 (PBST). This was followed by blocking each well with 5% dry milk and incubation at room temperature (RT) for 2 h. After washing three times with PBST, the denatured EDIII‐1‐4 produced in *E. coli* was added to plate 1, while lettuce‐produced EDIII‐1‐4 was added to plate 2 and incubated at RT for 2 h. After washing three times with PBST, the vaccinated rabbit serum was added to each plate. For the positive control, the polyclonal rabbit antiserum raised against EDIII peptide KFKVVKEIAETQHGT (Gottschamel *et al*., 2016) was used while for the negative control, serum obtained from an unvaccinated rabbit was used. All serum samples were applied as a dilution series, starting from a 1 : 50 dilution, then 1 : 100, 1 : 200, etc. The plates were incubated at RT for 2 h followed by washing three times with PBST. Thereafter, a goat anti‐rabbit polyclonal antibody conjugated to horse radish peroxidase (HRP) (DAKO; Glostrup, Denmark) was added to each well at a dilution of 1 : 1000. The plates were incubated for 1 h followed by washing. This was followed by adding the substrate made of OPD tablets (O‐Phenylenediamine dihydrochloride, DAKO; Glostrup, Denmark) plus 5% hydrogen peroxide (H_2_O_2_). The plates were observed for 15 min for colour change followed by adding 0.05 μL of the stop solution (1 mm H_2_SO_4_). The plates were read using a spectrophotometer (TECAN, Genios, Boston, MA) at an optical density of OD_490_. A Student's *t*‐test was used in statistical analysis.

### *In vitro* gastrointestinal digestion analysis

The harmonized, static *in vitro* digestion model (Minekus *et al*., 2014) was performed with fresh leaves from the lettuce plant line S12‐PN‐EDIII‐1‐4. The plant material was cut into pieces of 5 mm^2^ and a total amount of 2 g plant material per reaction was incubated under shaking in a water bath at 37 °C. The model consists of three stages: stage I ‐ oral phase: buffer (simulated salivary fluid: 15.1 mm KCl, 3.7 mm KH_2_PO_4_, 13.6 mm NaHCO_3_, 0.15 mm MgCl_2_(H_2_O)_6_, 0.06 mm (NH_4_)_2_CO_3_, 1.5 mm CaCl_2_(H_2_O)_2_, 1.1 mm HCl, pH 7), 0.44 U salivary α‐amylase (Sigma‐Aldrich, St. Louis, MO), 2 min reaction time; stage II ‐ gastric phase: buffer (simulated gastric fluid: 6.9 mm KCl, 0.9 mm KH_2_PO_4_, 25 mm NaHCO_3_, 47.2 mm NaCl, 0.12 mm MgCl_2_(H_2_O)_6_, 0.5 mm (NH_4_)_2_CO_3_, 0.15 mm CaCl_2_(H_2_O)_2_, 15.6 mm HCl, pH 3), 90 U porcine pepsin (Sigma‐Aldrich), 2 h incubation time; and stage III ‐ duodenal stage: buffer (simulated intestinal fluid (6.8 mm KCl, 0.8 mm KH_2_PO_4_, 85 mm NaHCO_3_, 38.4 mm NaCl, 0.33 mm MgCl_2_(H_2_O)_6_, 0.6 mm CaCl_2_(H_2_O)_2_, 8.4 mm HCl, pH 7), porcine pancreatin (4340 U protease; Sigma‐Aldrich) and 10 mm porcine bile extract (Sigma‐Aldrich), 2 h incubation time. Reactions in samples from all phases were stopped and neutralized by mixing with a TP extraction buffer containing protease inhibitors, as described above and immediately snap frozen in liquid nitrogen. Total protein was extracted as described above and subjected to Western Blot analysis.

## Author contributions

AvE: wrote the manuscript, helped with experiments. JG: this study was part of her PhD project; she carried out plant‐related experiments and the GI tract assay and wrote the manuscript. RB: was an external co‐supervisor to JG and involved in project planning and trouble‐shooting and contributed to writing. KEAH: performed the rabbit study, sample collection and contributed to the writing. HMM: conducted immunogenicity analysis and contributed to the writing. HD: gave advice about plant‐made oral vaccines to JLC and contributed to manuscript writing. JLC: was the project leader and co‐supervisor to JG. JLC designed the study, followed up the study through the whole project period and contributed considerably to the manuscript preparation.

## Conflict of interest

There is no conflict of interest for the current study.

## Supporting information


**Figure S1** Quantification of EDIII‐1‐4 accumulation in transplastomic lettuce plants.
