# Supplementary material for: Production of tetravalent dengue virus envelope protein domain III based antigens in lettuce chloroplasts and immunologic analysis for future oral vaccine development
Source: Plant Biotechnol J. 2019 Feb 19;17(7):1408–17. doi: 10.1111/pbi.13065 (PMC6576073; doi:10.1111/pbi.13065)
Supplement: Supplementary file 1 — Figure S1 Quantification of EDIII‐1‐4 accumulation in transplastomic lettuce plants. [file PBI-17-1408-s001.docx]

**Supplementary Figure 1:**


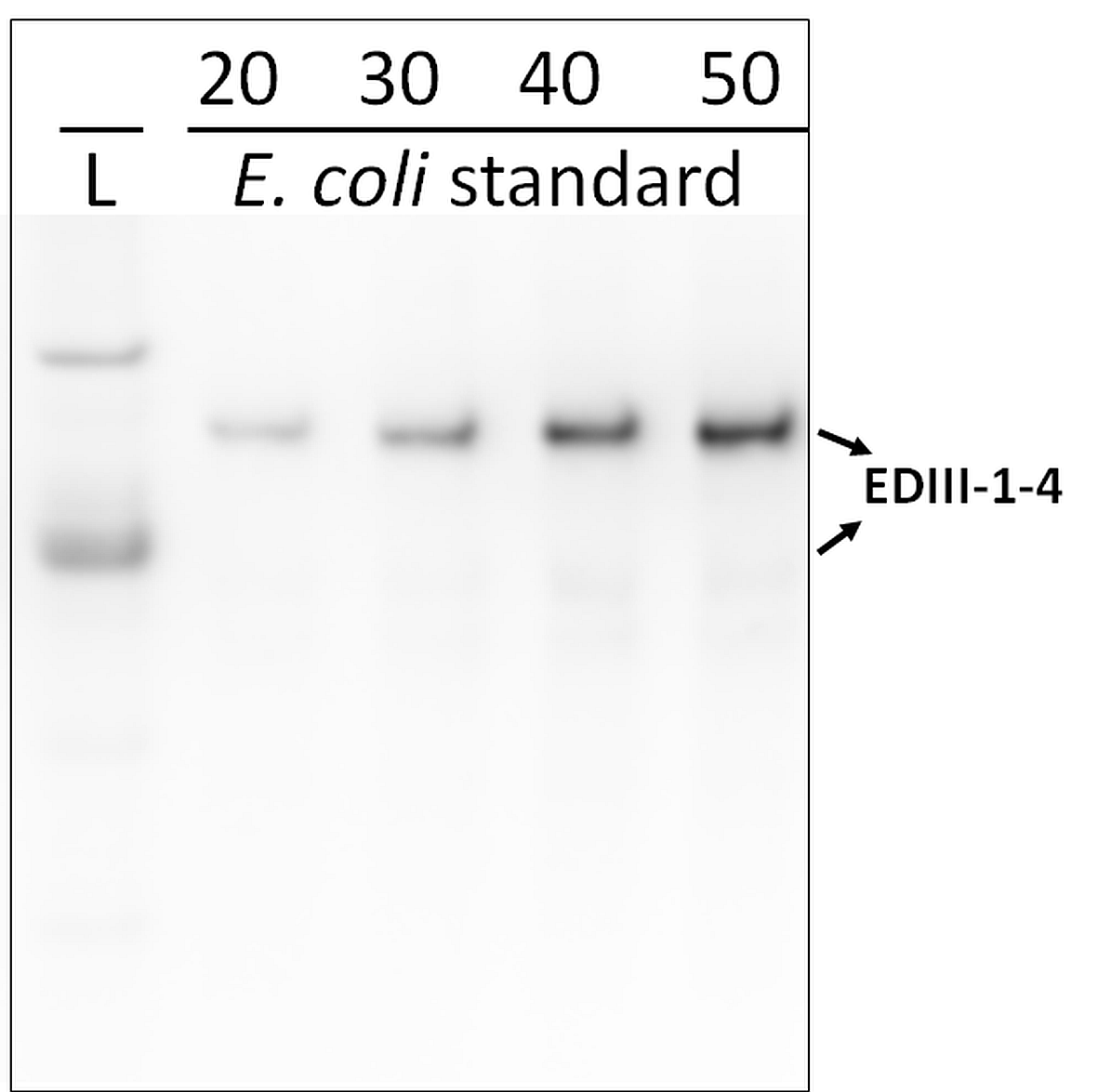


**Suppl. figure 1.** Quantification of EDIII-1-4 accumulation in lettuce. Western blot of a sample containing 1 µg of TP extract from lettuce line S12‑PN‑EDIII-1‑4 (L), compared with samples containing 20, 30, 40 and 50 ng of *E. coli*-produced EDIII-1-4, purified under denaturing conditions. *E. coli*-produced EDIII antigens run at a larger apparent molecular weight than plant-produced EDIII antigens, as was observed before (Gottschamel et al., 2016). The upper band in the lettuce sample results from non-specific binding of the antibody to an approximately 70 kDa plant protein of unknown identity (Gottschamel et al., 2016).
